# Dietary Modulation of Bacteriophages as an Additional Player in Inflammation and Cancer

**DOI:** 10.3390/cancers13092036

**Published:** 2021-04-23

**Authors:** Luigi Marongiu, Markus Burkard, Sascha Venturelli, Heike Allgayer

**Affiliations:** 1 Department of Experimental Surgery—Cancer Metastasis, Medical Faculty Mannheim, Ruprecht-Karls University of Heidelberg, Ludolf-Krehl-Str. 13-17, 68167 Mannheim, Germany; Luigi.marongiu@medma.uni-heidelberg.de; 2 Department of Biochemistry of Nutrition, University of Hohenheim, Garbenstr. 30, 70599 Stuttgart, Germany; markus.burkard@uni-hohenheim.de; 3 Department of Vegetative and Clinical Physiology, University Hospital of Tuebingen, Otfried-Müllerstr. 27, 72076 Tuebingen, Germany

**Keywords:** Phage, bacteriophages, diet, infection, colorectal, cancer, nutrition

## Abstract

**Simple Summary:**

The role and function of bacteriophages (phages) in the intestine, its health and microbial homeostasis has been underestimated so far. This interdisciplinary review highlights the effect of dietary compounds on phages and puts this into perspective with putative contributions of phages to gastrointestinal diseases, specifically inflammation, infection, and cancer. The review discusses novel fields of opportunities in this context. These include, but are not limited to, perspectives how a better understanding of modulating the activity of specific phages by particular nutritional components may contribute to reorganizing the microbial network, thus supporting in the combat, or even prevention, of inflammation or even cancer in the gut.

**Abstract:**

Natural compounds such as essential oils and tea have been used successfully in naturopathy and folk medicine for hundreds of years. Current research is unveiling the molecular role of their antibacterial, anti-inflammatory, and anticancer properties. Nevertheless, the effect of these compounds on bacteriophages is still poorly understood. The application of bacteriophages against bacteria has gained a particular interest in recent years due to, e.g., the constant rise of antimicrobial resistance to antibiotics, or an increasing awareness of different types of microbiota and their potential contribution to gastrointestinal diseases, including inflammatory and malignant conditions. Thus, a better knowledge of how dietary products can affect bacteriophages and, in turn, the whole gut microbiome can help maintain healthy homeostasis, reducing the risk of developing diseases such as diverse types of gastroenteritis, inflammatory bowel disease, or even cancer. The present review summarizes the effect of dietary compounds on the physiology of bacteriophages. In a majority of works, the substance class of polyphenols showed a particular activity against bacteriophages, and the primary mechanism of action involved structural damage of the capsid, inhibiting bacteriophage activity and infectivity. Some further dietary compounds such as caffeine, salt or oregano have been shown to induce or suppress prophages, whereas others, such as the natural sweeter stevia, promoted species-specific phage responses. A better understanding of how dietary compounds could selectively, and specifically, modulate the activity of individual phages opens the possibility to reorganize the microbial network as an additional strategy to support in the combat, or in prevention, of gastrointestinal diseases, including inflammation and cancer.

## 1. Introduction

The impact of the gut bacteriome on the human physiology is currently being investigated and seems to have a significant influence on the development and treatment of various diseases. Collectively, the over one thousand bacterial species residing in the human gut encode 3.3 million genes, expanding the human genome 150 times over [1]. Several studies have demonstrated that microorganisms present in the human gut (the gut microbiome) modulate human physiology at different levels. Intestinal bacteria not only metabolize polysaccharides that would be otherwise indigestible [2], but also regulate peristalsis [3], help to keep a proper intestinal morphology as it has been shown in a gnotobiotic pig model [4], maintain the integrity of the intestinal barrier [5,6,7], attenuate inflammation [8,9], reduce the virulence of pathogenic species [10], and even influence the action of anticancer drugs [11]. Although it has been proposed to consider the intestinal microorganisms as symbionts rather than simple commensal species [12], our understanding of the dynamics underlying the interactions between host and gut microbiome is still limited [13,14].

Bacteriophages (or phages for short) represent a significant modulator of the gut microbiome [15]. By definition, phages infect bacteria, but more and more data highlight the interrelation between eukaryotic cells and bacterial viruses. Phages can interact directly with the human body since they can translocate inside eukaryotic cells [16] and activate the immune system, exacerbating ongoing colitis symptoms and boosting the antibacterial response [17]. It has recently been proposed to consider phages as human pathogens [18].

In the last few years, phages have become a crucial topic in the medical and microbiological fields because these viruses can be used as a treatment of bacterial infections in the context of the rising problem of antibiotic resistance [19,20,21]. As our understanding of phage biology increases, the applications of phage therapy also expand. Phages have been applied to treat bacterial infections ever since their discovery, and phage therapy is becoming more and more popular in fields ranging from dentistry to medical microbiology [22,23,24,25]. For example, phages are currently being evaluated to fight infections in poultry that are still an economic and health issue [24]. Recent studies suggest that phages can also be applied in antiviral and anticancer therapies. For instance, it has been proposed that phage T4 might be used as a co-treatment for COVID-19 because this phage reduces the immune response, which is an important contributor to the fatality associated with this disease [26]. Furthermore, it has been shown that phages bind to cancerous cells and reduce the size of the tumor mass in different mouse models [17,27,28], opening the possibility of phage-mediated oncolytic virotherapy.

Diet can influence the gut microbiome, and it is actively used as an intervention to reduce the risk of developing diseases [29]. Particular components have been shown to be of benefit in the treatment of even severe disease conditions up to cancer. For example, in own previous studies, it was demonstrated that the plant-derivatives curcumin and artesunate inhibit tumor cell invasion and metastasis, at least in part via regulating the expression of proteolytic enzymes, the molecular cascades involving transcriptional factors and microRNAs, respectively [30,31,32,33]. However, there is a lack of studies describing how dietary compounds impact microorganisms in general and phages in particular. Seminal studies in the 1950 s demonstrated the antiviral activity of tannins, which are contained in popular beverages such as tea and coffee, and of acerin, the active component of maple fruit [34,35]. Especially, tea showed broad antimicrobial activity, including inactivation of phages [36]. It is also known that essential oils have antibacterial and antiviral properties as well as anti-inflammatory and regenerative activities [37]. Nevertheless, gaining experimental knowledge on the influence of dietary compounds on phages as modulators of microbiota has not yet been in focus of attention in the research community.

Most of the studies related to the effect of dietary compounds on phages have been focusing on human viruses associated with gastroenteritis. Phages have been used as surrogates for viruses that cannot be easily cultivated, such as norovirus, rather than for studying bacterial virus biology as such. Also, most of the bacteriophage studies so far have been limited to phages infecting *Escherichia coli* (coliphages). Nonetheless, *E. coli* plays an essential role in human health since certain strains of this species, known as Shiga toxin-producing *E. coli* (STEC), are widespread food-borne pathogens. The most prevalent STECs are O157, O26, O45, O103, O111, O121, and O145. These seven serotypes induce diseases ranging from acute diarrhea to hemorrhagic colitis and fatal hemolytic syndrome [38,39,40].

The main STEC derived virulence factor is shiga-like toxin (Stx), which is encoded by the prophages 933 J (*Stx1*) and 933 W (*Stx2*) [41,42]. Upon activation, these prophages express *Stx*, and they can horizontally spread this gene by transduction [43,44]. Genotoxins, such as cytolethal distending toxins and colibactin, are considered cancer risk factors and can be found in pathogenic *E. coli* strains [45]. Interestingly, many natural compounds have been shown to be bactericidal against pathogens [46], and to suppress the biological activity of toxins, including the cholera and ricin toxins [46,47,48,49]. Peas showed to bind with high efficiency Stx, acting as toxic-scavengers, whereas beans can reduce the intake of Stx [50].

We speculate that a better understanding of how phages are activated or inhibited in the human gut might be pivotal in modulating the intestinal microbiome, e.g., to counteract bacterial infections, inflammatory conditions, and even carcinogenesis and cancer progression. Such indirect antibacterial activity is a particularly relevant feature in light of the urgent need to identify alternatives and additional strategies to antibiotics to defeat (therapy-resistant) bacterial infections. The present review will summarize the current knowledge on the effect of dietary compounds on phages, their activity, and infectivity.

## 2. Interactions between Phages and Bacteria in the Gut Microbiome

**Figure 1:**
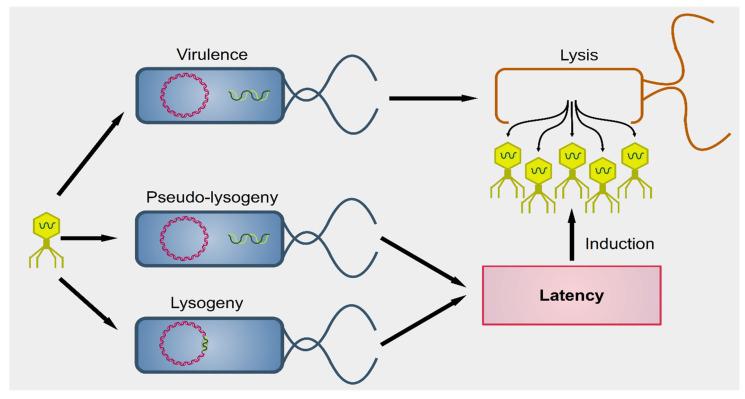
Outcomes of phagial infection of bacteria. A virulent phage (yellow particle on the left) can infect a bacterium (in blue). The replication of the phage leads to lysis of the host cell, releasing the viral progeny (yellow particles on the right). Alternatively, some viral species known as temperate can establish an additional step known as latency. The phagial genome can remain independent from that of the bacterium (pseudo-lysogeny) or become integrated into the host’s genome (lysogeny). In both cases, the viral expression is kept to a minimum and there is no virion production until several cellular conditions are met. Upon induction, temperate phages enter the lytic pathway and determine the lysis of the host.

Phages were first described by the French-Canadian Félix d’Hérelle, of Institute Pasteur in 1917, who also defined the term ‘bacteriophage’ (“eater of bacteria”). As a first, pioneering phage-based therapy, he applied bacteriophages to treat *Shigella* infections in soldiers, establishing what became known as phage therapy [51,52,53]. Phages can be subdivided into two groups: virulent (lytic) and temperate (lysogenic) (Figure 1) [54]. Lytic viruses start the replication process soon after the infection of the bacterial host. Once the progeny virions have assembled in a sufficient number (the burst size), the cell bursts open, releasing the new phages in the surrounding environment. Lysogenic phages have an additional phase: they can integrate as prophages in the bacterial chromosome and undergo a latency period where only a viral transcription suppressor is produced actively. In particular contexts, such as bacterial starvation or DNA damage, the suppression control is relieved, and the prophage enters the lytic phase. Conversely, in the presence of a high number of infected bacteria, phages exit the lytic phase and initiate lysogeny [55]. Both virulent and temperate phages modulate the bacterial population through lysis.

Phages can also modulate the bacterial population, indirectly. It is well known that bacteria must undergo a fierce competition within each ecological niche, and, therefore, some species have developed virulence factors to improve their chances of survival [56]. Moreover, the microbial competition is complex and difficult to predict. For instance, *Lactobacillus delbrueckii* and *L. rhamnosus* inhibit *E. coli* O157, but *L. plantarum* suppresses the commensal strains of *E. coli* but not O157, and *L. paracasei* does not constrain *E. coli* at all [57]. In addition, the suppression of one species might cause the unexpected expansion of a species not apparently associated with the suppressed one. For instance, *E. coli* fosters the growth of *B. fragilis* but represses *B. vulgatus*. Knocking down *E. coli* by phage T4 is, therefore, followed by a contraction of the prevalence of *B. fragilis* and an increased growth of *B. vulgatus,* but also of *Proteus mirabilis* and *Akkermansia muciniphila* [58]. It is also known that commensal species can neutralize toxins, reducing the fitness of the pathogens. For instance, surface proteins of *L. plantarum* can neutralize Stx, reducing the cytotoxicity (and, thus, the fitness) of *E. coli* O157 [59]. Therefore, the alteration of even one species due to phagial predation can have drastic consequences for the microbiome.

Mounting evidence suggests that phages have access to eukaryotic (and human) cells [60]. Even though tissues are expected to be sterile, it has been known for decades that an ingestion of phage preparations during phage therapy is followed by a recovery of phages in human urine and blood within a few minutes from the administration [61,62]. This recovery implies that the viruses had somehow crossed the gastrointestinal barrier. Recent virome studies have identified genes belonging to phages in both blood and brain [63,64]. The circulation of phages in the peripheral blood has been named ‘phagemia’, but there is a lack of hard evidence for its actual existence in physiological conditions [65]. Furthermore, phages can be actively transported from one side to another of intestinal cells (transcytosis) via the Golgi network [16].

## 3. Effect of Dietary Compounds on Phages

**Figure 2:**
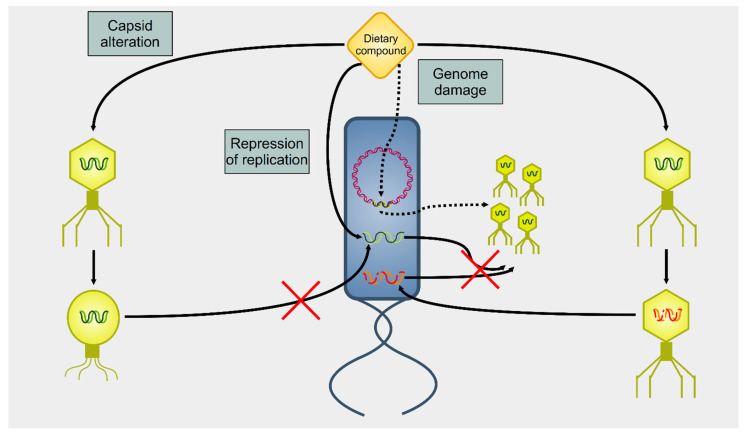
Summary of actions on phages on dietary compounds. There are three main mechanisms of action of dietary compounds upon phages. A dietary compound can modify the capsid, blocking the infectivity of the targeted phage (capsid alteration). Alternatively, dietary compounds can lead to the degradation of nucleic acids (genome damage). In this case, a phage can infect the host, but there will be neither lysis nor viral progeny. However, DNA damage to the host cell’s genome triggers the induction of prophages (dotted arrows). A final mechanism of action (repression of replication) involves an interference with the replication of the viral genome. Even in this case, there is infection, but no viral progeny is produced.

Several dietary compounds can alter the physiology of phages, as summarized in Figure 2. Although many studies showed a connection between nutrition and intestinal microbiome, there are only a few studies that deal with the effects of nutrition on the activity of phages. Seminal work in the 1960 s indicated that amino acids and vitamins had a different impact on the induction of prophage λ in *E. coli* [66]. For instance, the amino acid cysteine was an inducer, but its oxidized derivative cystine was not. About four decades later, it was shown that essential oils extracted from chamomile, lemongrass, cinnamon, and geranium could greatly reduce the infectivity of *E. coli* T7 and *S. aureus* SA, whereas others (such as angelica, cardamom, lime, and rosemary) affected only the former phage [67]. A recent study reported how different compounds could selectively activate some viruses but not others in bacterial growth and prophage-induction assays [68]. This study demonstrated how stevia, a natural sweetener obtained from the Brazilian shrub *Stevia rebaudiana* [69], could strongly induce prophages present in *Bacteroides thetaiotamicron* and *Staphylococcus aureus* but not in *Enterococcus faecalis*, whereas uva ursi (derived from the shrub plant *Arctostaphylos uva-ursi*), aspartame (a peptide), and propolis (a flavonoid) resulted in the opposite. These data indicate that dietary compounds can modulate the gut virome and, consequently, alter the gut bacteriome.

Experiments measuring the effect of dietary compounds on phage activity have been based on few classes of compounds, mainly polyphenols. These are molecules that contain one or more phenolic aromatic rings (benzenes with hydroxide moieties). Polyphenols can be subdivided into phenolic acid derivatives and flavonoids [70]. The former can, in turn, be subdivided into derivatives of either hydroxybenzoic acid (for instance, gallic acid) or cinnamic acid (for example, caffeic acid) [71]. Tea, the second most frequently consumed beverage after water, is a primary source for gallic acid [72]. Coffee, whose consumption is increasing worldwide [73], contains chlorogenic acid (a combination of caffeic acid and quinic acid) [71]. Tannic acid, which contains several hydroxybenzoic acid moieties, is particularly abundant in berries; soy is rich in isoflavonoids, such as genistein and daidzein [74]. The exact mechanism of action of these phenol-compounds is not entirely understood. Still, it is known that they can be beneficial for human physiology and have been used in folk medicine since millennia [75]. They are currently being investigated for their anticancer activity [76,77,78].

**Figure 3:**
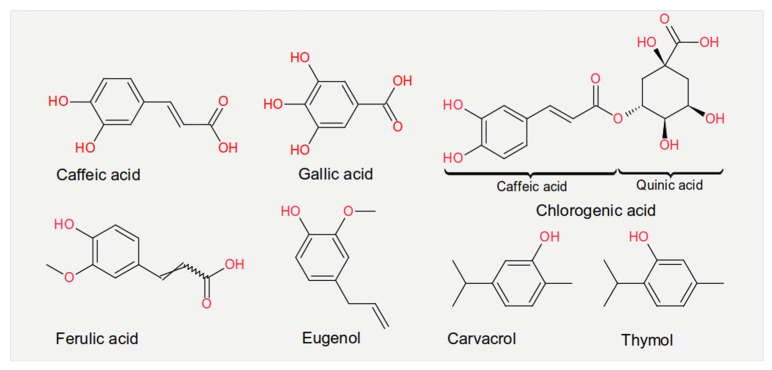
Chemical structures of the phenolic acids reported in the present review.

**Figure 4:**
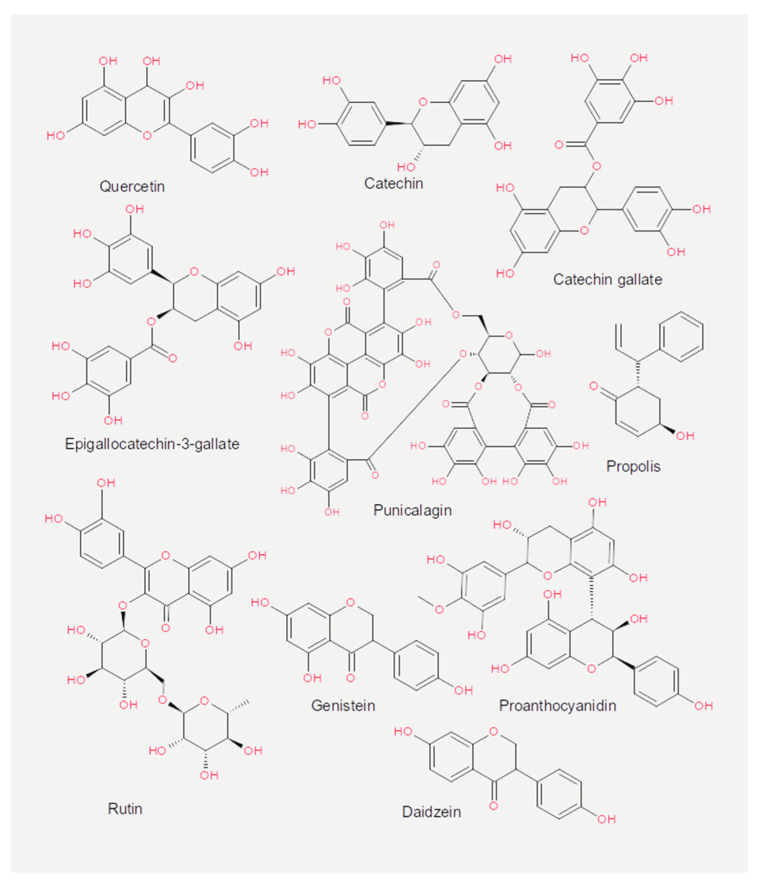
Chemical structures of the flavonoids reported in the present review.

**Figure 5:**
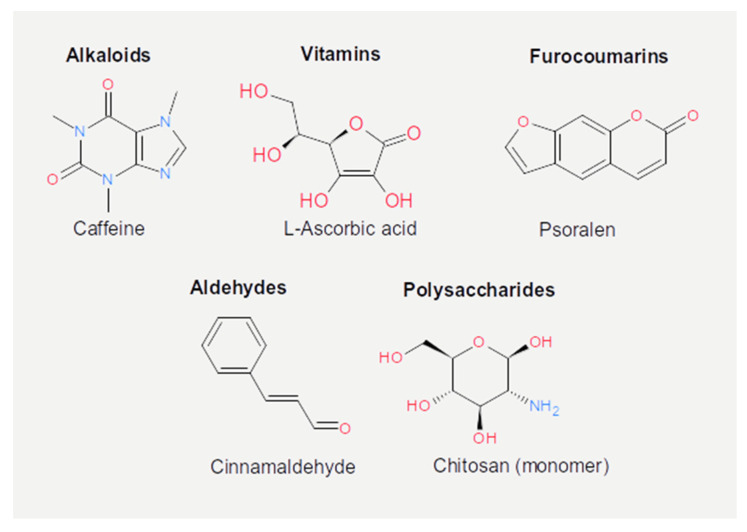
Chemical structures of the other active dietary compounds reported in the present review.

The chemical structure of the compounds discussed herein is shown in Figure 3, Figure 4 and Figure 5. A summary of the activities identified is given in Table 1. The most common outcome of exposure to a given nutrient is a loss of infectivity; this is measured by comparing the plaque-forming units (PFU) of a control and an exposed suspension (measured in mL) of phages. If the control and the exposed suspensions showed, for instance, 10^10^ and 10^9^ PFU/mL, then the reduction is said to be one log_10_. Herein, we will report the results using this notation.

### 3.1. Phenolic Acids

Roasted coffee, but not freshly brewed coffee, has been shown to induce the prophage λ in *E. coli* [79]. However, the λ progeny suffered from aberrant replication, and most of the resulting virions were not infective [110]. Therefore, one hypothesis to explain this is that the compounds produced during the roasting process of coffee beans, such as aliphatic carbonyls or volatile substances [73], can cause DNA damage that, in turn, initiates a stress response and the consequent induction of λ prophages. The DNA damage also would explain why the viral progeny, whose genome is a linear double-stranded DNA (dsDNA) molecule, displays a reduced level of infectivity.

Potatoes are commonly used as food worldwide, particularly in the Western diet [111,112]. Potato peel extracts (PPE) contain a mixture of polyphenols (e.g., gallic acid, chlorogenic acid, caffeic acid, and ferulic acid) and flavonoids (such as quercetin and rutin). Exposure of the *E. coli* O157 phages MS2 and Av-05 to 5 mg/mL of PPE for three hours in vitro resulted in a 2.8 and 3.9 log10 reduction, respectively [113]. Hence, Av-05 was more susceptible than MS2 to PPE exposure. The inhibitory mechanism was probably due to interference with the replication stage. Also, tomatoes contain different polyphenols, mainly in leaves and stems. Although the exact composition of these polyphenols varies, gallic acid and chlorogenic acid belong to the most prevalent. Exposure to 5 mg/mL of tomato leaf extract (TLE) for 12 h reduced the infectivity of both MS2 and Av-05; the magnitude of the reduction depended on the tomato subspecies: the *Pitenza* cultivar reduced the infectivity of MS2 and Av-05 by 3.8 and 5 log10, respectively, compared to 0.57 and 1.6 log10 obtained with the *Floradade* cultivar [80]. Even in this case, the inhibitory mechanism was supposed to be linked to viral replication.

Caffeic acid could also inhibit the cytotoxicity of Stx in a Vero-d2EGFP cell-based assay, in a process independent from the alteration of the induction of 933J and 933W [114]. Gallic and caffeic acids at low concentration (around 10^–6^ mg/mL) and tannins (0.5 mg/mL) reduced the infectivity of PL-1 (infecting *L. casei*) by 80–90% [81]. Others reported that both tannic (0.01–0.1 mg/mL) and gallic (0.1–0.4 mg/mL) acids had negligible action upon the infectivity of MS2, with a reduction that reached a maximum of 0.06 log_10_ [82].

*Zataria multiflora* is an aromatic plant native from Iran and Afghanistan that is rich in the monoterpenoids carvacrol (or cymophenol) and thymol [115]. A 0.03% *v*/*v* of *Z. multiflora* extracts were bacteriostatic for *E. coli* O157, but sub-inhibitory concentrations reduced the induction of 933 W, measured by quantifying the expression of *Stx2* [83]. Several other compounds, including several derivatives of gallic acid, showed antiviral activity in vitro measured with the MTT method and estimated by the inhibition of viral cytopathic effects [116].

### 3.2. Flavonoids

Flavonoids also belong to the class of polyphenols. In natural sources, they are usually mixed with other phenolic acids; thus, it is difficult to separate the former’s activity from that of the latter. Nevertheless, the active compound of cranberry juice is believed to be proanthocyanidin, a flavonoid [90]. In contrast, the active compounds of pomegranate juice extracts (PJE) were identified in punicalagin, a phenolic acid with antioxidant properties that could also inhibit the influenza virus [117,118]. Flavonoids are classified as antioxidants because they can react with, and remove from the cellular environment, the highly reactive superoxide anions (O_2_^−^) in a process known as scavenging [119]. Flavonoids include two products, catechin and genistein, with peculiar characteristics. Catechin is the basic block of tannins, found in fruit, tea, and wine; genistein is present in many medicinal plants.

Tea extracts were able to inactivate the *Salmonella* phages Felix 01 and P22, without affecting the growth of the bacterial host [84]. Exposure to 35 mg/mL of catechin for 24 h reduced the infectivity of the coliphage T4 by over two log_10_ in vitro, whereas the host did not show any reduction in population [86]. In addition, derivatives of catechins extracted from green tea could inhibit prophage induction. Epigallocatechin-3-gallate (EGCG) decreased the expression of *Stx1* but increased that of *Stx2* in *E. coli* O157 [87]. Since the expression of these two toxins is associated with the induction of 933 W in a germ-free mouse model [120], it needs to be assumed that, in this situation, EGCG is able to act as a virus inhibitor. The mechanism of action of EGCG involves the repression of the bacterial gene *recA* [87], an effector of the stress response that is central in the induction of 933 J, whereas the induction of 933 W relies on additional pathways not related to the expression of *recA*. This difference explains why only *Stx1* was reduced upon exposure to EGCG. This nutrient is believed to cause membrane damage that affects the growth of *E. coli* O157 and triggers stress response [87]. Other studies suggested that *Stx1* was still produced upon stimulation with EGCG and gallocatechin gallate (GCG), but the toxin’s extracellular release from *E. coli* O157 cultured at 37 °C for 24 h was hampered, probably due to both the galloyl and the hydroxyl moieties of these compounds [121].

Tannic acid is known to have antioxidant properties, since it can bind and remove singlet oxygen (^1^O_2_) from the cellular environment [122]. A 0.3% *w*/*v* solution of persimmon, a tannin, could induce a 3.13 log_10_ reduction in the infectivity of MS2. Electron microscopy confirmed that such exposure caused capsid denaturation [123].

Genistein and daidzein extracted from soybeans could protect the genome of phage φX174 from degradation induced by nitric oxide (NO) or peroxynitrite (ONOO^–^). Genistein was more effective than daidzein since a 25 µM solution of these dietary compounds protected about 75% and 45% of the viral φX174 DNA molecule confirmed by agarose gel electrophoresis, respectively [89]. This protection might be due to the scavenging properties of the flavonoids [124]. Genistein was also used to protect modified phages containing thymidine kinase derived from Herpes simplex virus during the delivery of this cytotoxin enzyme to tumor cells, thus increasing the targeted elimination of cancer cells [125].

Cranberries are fruits imported from North America and traditionally used by native Americans to treat bacterial infections. Investigations showed that cranberry juice could drastically reduce the growth of *E. coli* O157 in vitro [126]. Exposure for one hour to cranberry juice reduced the infectivity of the coliphages MS2 and φX174 by 1.67 and 1.22 log_10_, respectively, compared to the 0.05 and 0.29 log_10_ obtained by orange juice, 0.97 and 1.01 log_10_ obtained by grape juice, and 1.00 and 2.63 log_10_ obtained by purified proanthocyanidin [90,91]. Experiments with the coliphages T2 and T4 confirmed a complete and immediate loss of infectivity for these viruses when exposed to cranberry juice purchased from food shops [90,95]. Proanthocyanidin is also contained in blueberries; accordingly, exposure of MS2 to blueberry juice for 21 days induced a 6.32 log_10_ reduction in its infectivity when compared to incubation in phosphate buffered saline (PBS) [127].

In some studies, pomegranate and grape seed juices, which are rich in both flavonoids and phenolic acids, showed an antiviral activity. PJE at a 4 mg/mL concentration displayed a 0.12–0.32 log_10_ reduction upon MS2 infectivity in vitro [85,92]. This was in the same order of magnitude of other experiments carried out with pomegranate juice applied for 21 days, which showed a 0.14 log_10_ reduction in MS2 infectivity. Moreover, pomegranate juice diluted in PBS increased the inactivation to 1.84 log_10_ [127]. MS2 incubated in 1 mg/mL of grape seed extract (GSE) for two hours showed a 1.66 log_10_ reduction evaluated by plaque assay [93]. GSE also inhibited the growth of non-O157 *E. coli* serotypes, and GSE at a concentration of 4 mg/mL reduced the production of *Stx2* [83,94,128]. By comparison, pomegranate, grape, and orange juices showed lower, albeit still significant, reduction in phage infectivity in vitro [85,127]. In addition, grape seeds, which contain epicatechin, gallocatechin, GCG, and EGCG, could inactivate the cytotoxicity of Stx [114].

Su and colleagues suggested that cranberry juice in general, and proanthocyanidin in particular, inhibits the attachment phase of infecting phages in vitro, possibly via alterating the capsid [91]. This suggestion has been confirmed by electron microscopy analysis, which revealed that T4 treated with cranberry juice did not attach to their host [95]. Moreover, the feline calicivirus 9 showed structural modification of the capsid upon exposure to cranberry juice [91]. Likewise, apple juice, which is rich in procyanidins, increased the resistance of Vero cells against Stx [129].

Propolis (“bee glue”, a mixture of the saliva of honey bees with beeswax and plant exudates) contains flavonoids [130]. As mentioned above, it has been shown to specifically induce prophages in *E. faecalis* but not *B. thetaiotamicron* and *S. aureus* [68]. Brazil is the major producer of propolis and this natural substance can be classified according to its color. Green propolis induced a 3.0 log_10_ reduction in the infectivity of MS2 and 3.5 log10 in Av-08; red propolis was even more effective in reducing PFU, with a 4.2 and 4.0 log10 reduction for MS2 and Av-08 [96]. The main active molecule of red propolis is formononetin and the suggested mechanism of inhibition was alteration of the structure of the capsid [131].

### 3.3. Saccharides

Chitosan is a family of polysaccharides present in the exoskeleton of crustaceans and insects as well as in the cell wall of fungi. The members of this family are classified according to their molecular weight [132]. A 0.7% *w*/*v* solution of chitosan applied for three hours could decrease the infectivity of MS2 by up to 2.80 log_10_ (when using a molecule with a molecular mass of 53 kDa) and 5.16 log_10_ (when using 222 kDa). By increasing the concentration to 1%, only the 222 kDa form could completely inhibit MS2 [98,133,134]. Higher concentrations of both forms (1.5% *w*/*v*) were needed to achieve the inactivation of φX174, albeit the magnitude was much smaller than that of MS2 (0.94 log_10_). Chitosan was also active against *Bacillus thuringiensis* phage 1–97 A [99] and *Lactococcus lactis* phage c2 in vitro [100]. Furthermore, in vivo experiments with mice showed that chitosan was able to reduce Stx expression and the diffusion of induced 933 W progeny into the tissues, and to improve the lifespan of mice infected with enterohemorrhagic *E. coli* [101]. The mechanism of action was hypothesized to be a structural modification of the capsid [134,135,136]. Moreover, mutagenic effects of a sucrose-rich diet were reported by Dragsted et al. when investigating the colon of Big Blue rats, a specific strain of Fischer rats that carries 40 copies of the lambda phage on chromosome 4. In this study, a sucrose-rich diet resulted in an increase of mutational frequency in the DNA of these colons [137]. Lysozyme, which is widely distributed among prokaryotes and eukaryotes, is expressed by the R gene of phage lambda. Accordingly, the latter is called bacteriophage lambda lysozyme (LaL), and it has been shown to have bacteriolytic capabilities [138]. In contrast to other lysozymes, however, LaL differs regarding the cleavage of the glycosidic bond between N-acetylmuramic acid and N-acetylglucosamine of bacterial peptidoglycan. Duewel and colleagues showed that high concentrations of β(1→4) N-acetyl-D-glucosamine oligomers inhibit LaL but are not cleaved by the enzyme [138]. A similar observation of degrading peptidoglycans into fragments has also been reported for lysates of phage Vi II [139].

### 3.4. Essential Oils and Vitamins

Several essential oils show antibacterial and antioxidant activity, together with antiviral function [140]. For instance, oregano, thyme, cinnamon, and allspice (a berry from *Pimenta dioica* used commonly in the food industry) extracts, amongst others, can reduce the growth of *E. coli* O157 [141,142]. A 4% *v*/*v* solution of cinnamon oil, whose main component is cinnamaldehyde, inhibited the growth of *E. coli* O157 in vitro, but sub-inhibitory doses reduced the expression of *Stx2* and the release of viral progeny [72,94]. As in the case of EGCG, the interference over phage induction was accompanied by down-regulation of the effector of the stress pathway *recA*, but also of the quorum sensing (QS) (*qseB*, *qseC,* and *luxS*) and oxydative stress (*oxyR*, *soxR*, and *rpoS*) pathways, as well as the polynucleotide phosphorylase PAP I [94], which is also an inducer of 933 W [143]. These results suggest that cinnamon oil could interfere with 933 W induction as several overlapping levels. Furthermore, cinnamon oil disrupted *E. coli* O156 and *Pseudomonas aeruginosa* biofilms by interfering with the formation of the fimbriae, which are required to make inter-bacterial connections [72,94]. Oregano had a general suppressive action upon prophages, but the effect was stronger in *S. aureus* than in *E. faecalis* or *B. thetaiotamicron* [68]. Eugenol, which is rich in allspice and clove, reduced the induction of both Stx1 and Stx2, and inhibit the growth of *E. coli* O157 in vitro [144].

After a lag phase of few minutes, ascorbic acid (also known as vitamin C), reduced the infectivity of several phages: δA and φX174 (with a genome of ssDNA); T7, P22, D29, and PM2 (dsDNA); and MS2 (ssRNA) in vitro [88,102,103,104,105,106]. Supplementation of ascorbic acid with oxidants such as oxygen and hydrogen peroxide enhanced this effect, whereas antioxidants (for instance thiol compounds), nitrogen gas bubbling, or chelating agents suppressed it [102]. It was postulated that the autoxidation of ascorbic acid produced hydrogen peroxide that damaged the genome of the phages, even though Murata and colleagues found that hydrogen peroxide produced by autoxidation of ascorbic acid did not exert effects on activity of phage δA, in contrast to free radical intermediates [145]. Thus, the scavenging activity provided by thiols and chelating agents was hypothesized to reduce the damage on the viral genome, and the initial delay in the activity of ascorbic acid was interpreted as the time required to internalize this hydrogen peroxide inside the capsid [102]. Subsequent in vitro studies confirmed this hypothesis and showed that ascorbic acid caused the accumulation of nicks in both DNA and RNA genomes, with double-stranded genomes being less affected than single-stranded ones [88,103]. In these studies, the overlap of the nicks determined the formation of double strands breaks, which in fact sometimes appeared after the nicks as a result of the stochastic overlapping of the single-stranded lesions. Furthermore, these damages could be restored by the host’s cellular DNA repair system [88].

The oxidized form of vitamin C, dehydroascorbic acid, showed only very limited effects on phage activity and the amount of strand cleavages in ssDNA from phage δA was proportional to ascorbic acid concentration and incubation time. It was significantly increased by Cu2+ or hydrogen peroxide [102,146]. These DNA-damaging properties of the strong reducing combination of ascorbic acid with metal ions (especially Cu^2+^) [103] can have an impact on the phage population in the intestinal microbiome, but could also have implications in other fields such as the application of high-dose ascorbate in tumor patients. Towards this end, tumor entities like non-small-cell lung cancer and glioblastoma seem to be vulnerable towards the disruption of their intracellular iron metabolism and oxidative damage caused by the formation of hydrogen peroxide and hydroxyl radicals [147].

### 3.5. Other Compounds

There are very few studies investigating the impact on phages of molecules other than those listed so far. Pioneering work in the late 1950 s demonstrated how hydroquinone and pyrogallol (both derivatives of phenol but not polyphenols) reduced the infectivity of T coliphages [148]. Psoralens belong to the family of furocoumarins, photoactive polyphenols that can induce DNA damage. They are particularly abundant in the peel of limes [149]. Accordingly, a six-hour exposure with lime juice in vitro reduced the infectivity of MS2 by 1.3 log_10_ even in the absence of photoactivation [107].

Coffee contains not only caffeic acid but also caffeine, an alkaloid; coffee is a beverage on its own and the base for a plethora of soft-drinks [150]. High consumption of coffee and its derivatives has been suggested to confer an increased risk of colorectal cancer, due to its antimicrobial activity that disrupts the intestinal homeostasis [151]. Caffeine is able to induce *E. coli* phage φX174 in mitomycin treated *E. coli* cells [108]. Since caffeine is known to distort DNA and cause mutations [152], its activity is supposedly similar to caffeic acid in terms of inducing a stress signal that starts the lytic process.

Finally, even common salt used for meat preservation has been reported to exert effects on phage biology. Towards this end, a 2% *w*/*v* concentration of sodium chloride increased the expression of *Stx2*, as measured by immunoblotting, and the activation of the 933 W, as measured by plaque assay, in *E. coli* O157 [109].

### 3.6. In Vivo Studies

In contrast to a steadily increasing body of in vitro data that evaluates the interplay of diet or certain nutrients with bacteriophages as discussed in the chapters above, there are still only a few in vivo studies available. However, the possibility to modulate the microbiome by phage application is currently starting to attract more and more attention, especially in the field of inflammatory and malignant diseases. For instance, Zheng and colleagues covalently linked irinotecan-loaded dextran nanoparticles to azide-modified phages that were able to inhibit the growth of *Fusobacterium nucleatum* [153]. After i.v. or oral administration, these phage-guided irinotecan-loaded nanoparticles increased the chemotherapeutic efficacy in mice with colorectal tumors. In another study, a single injection of a lytic bacteriophage cocktail was effective as a rescue treatment for murine severe septic peritonitis, resulting in a significant improvement of the disease state without harming the microbiome [154]. Wild-type phage T4 and the according substrain HAP1, which is characterized by enhanced affinity to melanoma cells, were able to reduce lung metastasis of murine B16 melanoma cells by 47% and 80%, respectively [28]. Moreover, the modulation of the intestinal microbiome and metabolome was investigated using cognate lytic phages in gnotobiotic mice that were colonized with defined human gut commensal bacteria. This approach directly impacted susceptible bacteria, but phage predation also regulated additional bacteria via interbacterial interactions, yielding strong cascading net effects on the gut metabolome [58]. In a gnotobiotic pig model, it was shown that bacteria species are able to affect intestinal morphology as well as the expression of proinflammatory cytokines such as IL-1β and IL-6. Therefore, it can be hypothesized that a modulation of, e.g., neonatal bacterial colonization would have strong implications for a healthy development of the intestine [4]. On the other hand, intestinal inflammation and ulcerative colitis can be aggravated by high levels of certain bacteriophages that induce interferon-γ release [17]. Different phage cocktails (*ShigActive*™ [155] and *ListShield* [156]) have been shown to reduce shigella colonization of the murine gut and to decrease *Listeria monocytogenes* in the gastrointestinal tract, respectively. *ShigActive*™ was found to have comparable therapeutic effects to ampicillin but without the harmful effects on the gut microbiota exerted by the antibiotic [155]. *ListShield* was applied via oral gavage before mice were orally infected with *Listeria monocytogenes*. Consequently, *Listeria monocytogenes* concentrations were found to be reduced in the liver, spleen, and intestines when compared to controls. Even though, this phage therapy was as effective as the treatment with an antibiotic, it did not result in weight loss of the animals in contrast to infected controls and antibiotic-treated mice [156]. In another study, mice with antibiotic-induced perturbed microbiomes were treated with autochthonous virome transfer and viable phages were effective in reshaping the murine gut microbiota in a way that closely resembled the pre-antibiotic situation [157]. In vivo targeting of specific bacterial pathogens with recombinant or wildtype phages was also investigated for *Clostridium difficile* infections [158], Vancomycin-resistant *Enterococcus faecalis* infections [159], Crohn’s disease [160] and even for the attenuation of alcoholic liver disease [161]. The human Bacteriophage for Gastrointestinal Health (PHAGE) study and PHAGE-2 study demonstrated that an application of therapeutic doses of bacteriophages was both safe and tolerable [162,163,164]. The double-blinded, placebo-controlled crossover PHAGE trial with adults consuming bacteriophages for 28 days (32/43 participants finished the study) also demonstrated that bacteriophages are able to selectively decrease the amount of target organisms, without disrupting the gut microbiome globally [162]. In the randomized, parallel-arm, double-blinded, placebo-controlled PHAGE-2 study (68 participants, four weeks), it could be shown that adding supplemental bacteriophages (PreforPro) to the probiotic *Bifidobacterium animalis* subsp. *lactis* enhanced positive effects on gastrointestinal health [164]. Taken together, there is increasing evidence, in initial in vivo studies, for the high potential of treating different diseases with bacteriophages and for the ability to reshape the gut microbiome via tailored phage cocktails. Still, however, more in vivo studies are needed that investigate the complex interplay between diet and bacteriophages, especially in the context of the prevention and treatment of inflammatory diseases and cancer.

## 4. Conclusions

In conclusion, the present review shows that many dietary compounds and food ingredients display significant bioactivity with documented effects on phages. The dietary compounds discussed in this review can be consumed directly by diet (as in tea or coffee) or indirectly as food supplements. Still, most of the data reviewed and discussed herein pertain to *E. coli* as the, so far, best studied phage target in humans. Although being a common gut commensal, certain serotypes of this species pose a threat to public health regarding severe infectious and (in part systemic) inflammatory conditions as discussed in the Introduction. A few reports up to now even hypothesized particular *E. coli* strains such as those producing the genotoxin colibactin as potential tumor promoters [165], although their data were restricted to experimental models so far. In own genome data, we have preliminary evidence of sequences of particular *E. coli* strains in human colorectal carcinomas and even metastases (unpublished data based on genomes published in [166]).

The main activity of the dietary compounds discussed in the present review includes inhibitory effects on phages due to the alteration of the capsid, with subsequent reduction of infectivity. In other cases, the viral genome is being damaged, again inhibiting the infectivity of phages. However, some dietary compounds are able to induce (as with common salt or gallocatechin gallate) or repress (as with carvacrol) prophages. More importantly, a few dietary compounds display species-specific activities. For instance, stevia apparently acts as an inducer for *S. aureus* prophages, but not of those present in *E. faecalis,* whereas propolis displays the opposite actions.

**Figure 6:**
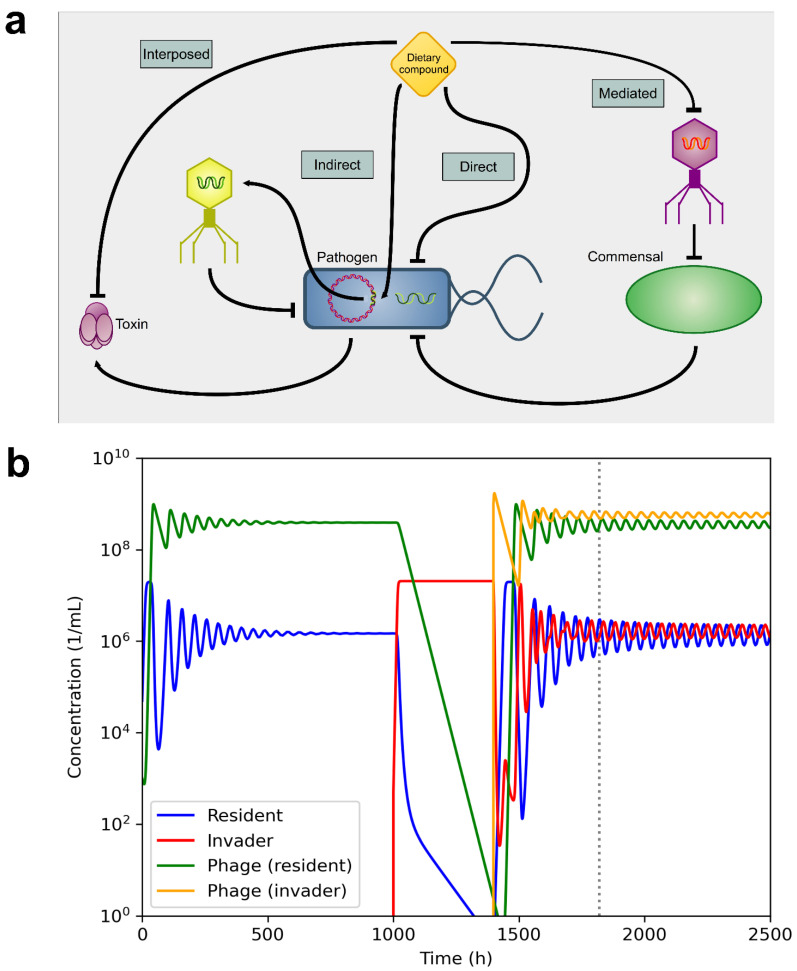
Overall impact of dietary compounds on infections. Dietary compounds can modify the bacterial population indirectly, based on the interaction between phages and bacteria, and by bacterial competition. (**a**) A dietary compound (nutrient) can interfere with the activity of a pathogenic bacterium at different levels by: inactivating a bacterial toxin (interposed inhibition); inducing a prophage already present in the invading bacterium, which then lyses the pathogen (indirect inhibition); acting bactericidal on invading species (direct inhibition); inactivating a lytic phage of an antagonist commensal species that, freed from the phagial burden, can compete with the pathogen (mediated inhibition). (**b**) Simulation of the interaction between bacteria and phages. The model considers the presence of a commensal bacterium (resident) and its phage. These reach an equilibrium where the number of cells or phages remains constant. A pathogenic bacterium (invader) will have virulence factors that favor its replication. As a result, it overgrows the commensal species. The activation of phages, namely through dietary-mediated induction of prophages, reduces the replication rate of the invader and re-establish, as a result, the commensal population. For the simulation, the parameters used were as follows. Carrying capacity: 2.2 × 10^7^. Maximum growth rate, 0.47 (commensal), and 0.72 (invader). Phage adsorption rate: 10^–9^, Phage lyse rate: 1.0. Phage burst size: 50. Particle loss rate: 0.05. Initial population of commensal bacteria: 50 000 cells. Amount of invader bacteria inoculated: 500 cells. Amount of phages: 1000 particles each. The model was implemented in *Julia* language using the *DifferentialEquations* package.

Overall, most of the dietary compounds reviewed here, with documented actions towards phages, showed a beneficial effect for the host by interfering with the activity of the pathogens at several levels. Thus, a number of concluding scenaria can be summarized for the putative benefit of nutrients (including the modulation of phages) to human patients and their microbiota (Figure 6A): Several nutritional compounds can directly affect the growth of microbial pathogens, but not that of commensals. Also, dietary compounds are able to inactivate particular toxins produced by pathogens, thus reducing fitness of the latter. More importantly, dietary compounds can inactivate virulent phages, modifying the overall equilibrium of the intestinal microbiome. As a result, phages targeting a commensal species that is a competitor to a pathogen can be removed from the niche. The commensal species will then expand and compete with the pathogen, again reducing the latter’s fitness. Finally, dietary compounds might induce prophages present in the pathogen, determining the hosts’ lysis and a wave of active virulent phages, which in turn reduce the pathogen’s population. Combining all of these inhibitory outcomes will reduce the pathogenicity of invading species and for example, help resolve infections or (chronic) inflammatory conditions.

To better understand how dietary compounds could selectively modulate bacterial infections, we carried out a simulation model (Figure 6b). This model shows that a pathogenic bacterium can wipe out a commensal species, but the selective induction of a prophage can then control the growth of the pathogen, reducing the virulence of the infecting species. The model suggests that it could be possible, in principle, to reorganize the microbial network to fight infections and further disease. Experimental data is required to assess the specificity of particular dietary compounds’ action to, effectively and safely, direct such attempts of specific reorganization.

Similar considerations as for phage-directed attempts to counteract infections and inflammatory conditions could be speculated for the field of carcinogenesis and cancer. Interfering with particular commensals within the intestinal microbiome by phages of different activities and properties, with the result of changing the intestinal microenvironment towards a more pro- or anti-carcinogenic condition, could be an exciting novel field of colorectal and other intestinal cancer research, and of treatment development. In parallel, more specific research on particular dietary compounds, chemical components, and associated modulation of phages that exert controllable, specific effects on the microbiome could open exciting new possibilities to interfere with intra-intestinal conditions in ways to foster anti-carcinogenic, more cancer-preventive environments.

## Figures and Tables

**Table 1:** Effect of the dietary compounds reported in the present review, stratified by chemical class and viral target. The principle of action is also reported, as far as known so far.

Nutrient	Class	Virus	Effect	Mechanism	References
Caffeic acid (or carbonyls)	Phenolic acids (or hydrocarbons)	λ	Prophage induction	Stress response to DNA damage	[79]
		Av-5, MS2	Infectivity reduction	Inhibition of replication	[80]
Gallic acid, chlorogenic acid	Phenolic acids	Av-5, MS2	Infectivity reduction	Inhibition of replication	[80]
Gallic acid	Phenolic acids	PL-1	Infectivity reduction	Interference to infection	[81]
		MS2	No effect	Unreported	[82]
Carvacrol, thymol	Phenolic acids	933 W	Prophage suppression	Unreported	[83]
Tea extracts	Phenolic acids or flavonoids	Felix 01 and P22	Infectivity reduction	Unreported	[84]
Pomegranate juice (punicalagin)	Phenolic acids or flavonoids	MS2	Infectivity reduction	Interference to infection (Capsid denaturation?)	[85]
Catechins	Flavonoids	T4	Infectivity reduction	Unreported	[86]
EGCG	Flavonoids	933 J	Prophage suppression	Repression of *recA*	[87]
GCG	Flavonoids	933 W	Prophage induction	Stress response to ROS	[88]
Genistein, daidzein	Flavonoids	φX174	Genome protection	Scavenging	[89]
Proanthocyanidin	Flavonoids	MS2, φX174	Infectivity reduction	Capsid denaturation	[90,91]
PJE	Flavonoids	MS2	Infectivity reduction	Capsid denaturation	[92]
GSE	Flavonoids	MS2	Infectivity reduction	Interference to infection (Capsid denaturation?)	[93]
		933 W	Prophage suppression	Unreported	[94]
Cranberry juice	Flavonoids	T2, T4	Infectivity reduction	Capsid denaturation	[95]
Propolis	Flavonoids	Unreported	Prophage induction or suppression	Unreported	[68]
Red propolis (formononetin)	Flavonoids	MS2, Av-08	Infectivity reduction	Interference to infection (Capsid denaturation?)	[96]
Cinnamaldehyde (cinnamon)	Essential oil (aldehydes)	933 W	Prophage suppression	Repression of *recA*	[94,97]
Oregano	Essential oil	Unreported	Prophage suppression	Unreported	[68]
Chamomile, lemongrass, cinnamon	Essential oils	T7, SA	Infectivity reduction	Unreported	[67]
Chitosan	Polysaccharide	MS2, φX174	Infectivity reduction	Capsid denaturation	[98]
		1–97 A	Infectivity reduction	Capsid denaturation	[99]
		c2	Infectivity reduction	Capsid denaturation	[100]
		933 W	Infectivity reduction	Unreported	[101]
Ascorbic acid	Vitamin	δA, φX174, T7, P22, D29, PM2, MS2	Infectivity reduction	Genome damage	[88,102,103,104,105,106]
Psoralen	Furocoumarins	MS2	Infectivity reduction	Unreported	[107]
Caffeine	Alkaloids	φX174	Prophage induction	Stress response to DNA damage	[108]
Sodium chloride	Salt	933 W	Prophage induction	Unreported	[109]
